# Evaluation and application potential of an accelerometer-based collar device for measuring grazing behavior of dairy cows

**DOI:** 10.1017/S1751731118003658

**Published:** 2019-02-08

**Authors:** J. Werner, C. Umstatter, L. Leso, E. Kennedy, A. Geoghegan, L. Shalloo, M. Schick, B. O’Brien

**Affiliations:** 1 Teagasc, Animal & Grassland Research and Innovation Centre, Moorepark, Fermoy Co. Cork, P61 C997, Ireland; 2 Institute for Agricultural Engineering, University of Hohenheim, 70599 Stuttgart, Germany; 3 Research Division Competitiveness and System Evaluation, Agroscope, 8356 Ettenhausen, Switzerland; 4 Department of Agricultural, Food and Forestry Systems, University of Florence, 50145 Firenze, Italy; 5 Division Animal Husbandry & Dairy Production, Strickhof, 8315 Lindau, Switzerland

**Keywords:** validation, sensor technology, RumiWatch, MooMonitor+, pasture management

## Abstract

The commercially available collar device MooMonitor+ was evaluated with regards to accuracy and application potential for measuring grazing behavior. These automated measurements are crucial as cows feed intake behavior at pasture is an important parameter of animal performance, health and welfare as well as being an indicator of feed availability. Compared to laborious and time-consuming visual observation, the continuous and automated measurement of grazing behavior may support and improve the grazing management of dairy cows on pasture. Therefore, there were two experiments as well as a literature analysis conducted to evaluate the MooMonitor+ under grazing conditions. The first experiment compared the automated measurement of the sensor against visual observation. In a second experiment, the MooMonitor+ was compared to a noseband sensor (RumiWatch), which also allows continuous measurement of grazing behavior. The first experiment on *n* = 12 cows revealed that the automated sensor MooMonitor+ and visual observation were highly correlated as indicated by the Spearman’s rank correlation coefficient (*r*_*s*_) = 0.94 and concordance correlation coefficient (CCC) = 0.97 for grazing time. An *r*_*s*_-value of 0.97 and CCC = 0.98 was observed for rumination time. In a second experiment with *n* = 12 cows over 24-h periods, a high correlation between the MooMonitor+ and the RumiWatch was observed for grazing time as indicated by an *r*_*s*_-value of 0.91 and a CCC-value of 0.97. Similarly, a high correlation was observed for rumination time with an *r*_*s*_-value of 0.96 and a CCC-value of 0.99. While a higher level of agreement between the MooMonitor+ and both visual observation and RumiWatch was observed for rumination time compared to grazing time, the overall results showed a high level of accuracy of the collar device in measuring grazing and rumination times. Therefore, the collar device can be applied to monitor cow behavior at pasture on farms. With regards to the application potential of the collar device, it may not only be used on commercial farms but can also be applied to research questions when a data resolution of 15 min is sufficient. Thus, at farm level, the farmer can get an accurate and continuous measurement of grazing behavior of each individual cow and may then use those data for decision-making to optimize the animal management.

## Implications

Monitoring feed intake behavior of cows is important for determining the health status of the cow and the onset of estrus as well as the feed budget for the cow. As herd sizes increase and the availability of labor decreases, the monitoring of each individual cow may be supported by automated sensors. There are limited sensors available for measuring grazing behavior. The validation study conducted here for the MooMonitor+ showed a very high accuracy in measuring grazing behavior. These results proved that the sensor can be applied on the farm and can assist the farmer in managing the herd.

## Introduction

Sensor technology has developed and improved rapidly in recent years. Technological systems have advanced to measure a broad range of parameters, such as acceleration, temperature and pH, which has had a positive impact on the application range of sensors. Technical progress can be observed in different areas of the agricultural sector. In dairy farming, the application of sensor systems should assist farmers to manage larger animal groupings. With higher numbers of cows and less available time per animal, farmers in their daily routine may be supported in decision making by an automated continuous measurement of parameters to maintain good animal management. Animal management in a dairy system involves ensuring health and welfare of the animals; reacting to certain events in the animal reproductive cycle and improving efficiency in feed conversion to the animal product, for example, milk.

The parameter ‘feed intake behavior’ is one of the best indicators of health and welfare of dairy cows. Bareille *et al*. (2003) found that feed intake decreased at the initial stages of ketosis and mastitis. These findings were underlined by a study of Gonzalez *et al*. (2008), where ketosis, lameness and mastitis all had a negative impact on feeding behavior. Mastitis is recognized as one of the major causes of reduced profit (Kossaibati and Esslemont, 1997). Thus, early detection of this emerging disease would not only be beneficial to the animal through earlier treatment, but would also improve farm profitability. As well as a potential indicator of health issues, feeding behavior and especially rumination, may be a valuable parameter in determining the status of the reproductive cycle of dairy cows. Rumination time may be used to predict calving (Soriani *et al*., 2012) as well as determining an estrus event (Mahmoud *et al*., 2017). A study by Stangaferro *et al*. (2016) successfully linked the identification of metabolic disorders with rumination and physical activity. Further, the knowledge generated on detailed grazing behavior may also assist in efficient grazing management. Based on results of a study by Chilibroste *et al*. (1997), the grazing time is affected by the presence of indigestible material in the rumen as well as the degree of starvation before the actual grazing. In addition, it is identified that the rumination time decreases with less available material in the rumen to digest (Kennedy *et al*., 2009). These facts may be used to optimize the correct grass allocation to the cows, considering grazing and rumination time of cows as measured by these automated sensors.

The importance of sensor systems is also increasing as the availability of labor decreases on family farms due to increases in scale and less frequent involvement of adult children in the family (Barkema *et al*., 2015). As farm size increases, there is a general tendency for the businesses to involve more hired labor rather than experience-based family workers (Eastwood *et al*., 2016). These facts all support the inclusion of sensor technology as a decision support tool on farms.

The MooMonitor+ (Dairymaster, Tralee, Ireland) is a collar device with an integrated 3-axis accelerometer designed for heat detection and has been commercially available since 2014. Subsequently to further development, this device may have the capability to record grazing and rumination time accurately. However, an independent evaluation of the suitability of the sensor device for accurate measurement of grazing behavior was not published yet. Thus, the primary objective of this study was to validate this automated sensor against visual observation with regard to accuracy in monitoring grazing behavior. A secondary objective was to compare the MooMonitor+ with the RumiWatch noseband sensor as this device allows continuous observation as well and has been previously validated for measuring feeding behavior in research (Werner *et al*., 2017). Finally as a third objective, the application potential of these used sensors and sensors in literature is discussed with regard to measurement of grazing behavior in both scientific and commercial scenarios.

## Material and methods

### Experiment 1

The first experiment was conducted between 10 and 19 May 2016 on a group of 18 spring calving dairy cows on the Teagasc research farm in Moorepark, Fermoy, Ireland. The data of six of these cows were analyzed to align times between the visual observations and the automated sensor (MooMonitor+). Due to the technical specification of the internet connection at the research farm, the timestamp on the base station linked with the sensors was not correct. Therefore, to investigate the correct time stamp, data of six cows were used to validate the automated sensor against human observer. Those data were excluded afterwards from the experimental data set.

#### Animals and treatments

Twelve cows were used for validation. This group consisted of six Jersey crossbred (JEX) and six Holstein-Friesian (HF) cows. There were four primiparous and eight multiparous cows involved, the range of lactation was from 2 to 6. The mean body condition score (BCS)± SD was 2.8±0.2 (based on a 1 to 5 scoring system with 0.25 increments; Edmonson *et al*., 1989). Average BW was 477±65 kg. The milk yield was 22.5±4.5 kg/cow per day over the experimental period and average days in milk (DIM) was 91±12 at the beginning of the experiment. All cows followed a similar milking schedule, being milked twice daily at 7:00 and 14:30 h with approximately 1.5 to 2.0 h away from the paddock during each milking. Cows were fed with only grass on the paddocks with no additional supplementation of concentrate. A fresh allocation of pasture was provided after each milking. Pre- and post-grazing grass heights were measured daily using a rising plate meter (diameter 355 mm and 3.2 kg/m^2^; Jenquip, Fielding, New Zealand). Pre-grazing heights and post-grazing heights were 11.9±2.5 cm and 4.5±0.8 cm, respectively, during the experimental period. These values represented a non-restrictive grazing management strategy in Ireland, where cows received a daily herbage allowance of 16.3±2.6 kg dry matter (DM)/cow per day, measured above 3.5 cm sward height, on average during the experimental period (McCarthy *et al*., 2013). The chemical composition was analyzed once weekly resulting in an average DM content of 15%, an average CP content of 22% and an average content of NDF and ADF of 41% and 23%, respectively.

#### Experimental design

Grazing and rumination time data were collected by visual observation according to a 1-min scan sampling protocol, similar to the method used in the study of Büchel and Sundrum (2014) and by the MooMonitor+. Two previously trained observers were monitoring 18 cows in total (12 cows for validation; 6 cows for time alignment). The cows were divided into six subsets with three cows each for the purpose of observation. Each subgroup was observed by each observer on three occasions over 6 days (Table 1). Observations took place over 2-h periods between dawn (05:00) and dusk (21:00) excluding milking times from 07:00 to 09:00 h and 14:00 to 17:00 h. After the first 3 days, the times were changed to cover the full range of daylight hours within the days.

**Table 1:** Experimental protocol for cow grazing and rumination data collection by visual observation

		Cow number				Cow number		
Days	Time	1	2	3	Time	4	5	6
Observer 1	09:00 to 11:00	1, 2, 3	7, 8, 9	16, 17, 18	05:00 to 07:00	4, 5, 6	10, 11, 12	16, 17, 18
	12:00 to 14:00	4, 5, 6	10, 11, 12	13, 14, 15	11:00 to 13:00	1, 2, 3	7, 8, 9	13, 14, 15
	17:00 to 19:00	1, 2, 3	7, 8, 9	16, 17, 18	19:00 to 21:00	4, 5, 6	10, 11, 12	16, 17, 18
Observer 2	09:00 to 11:00	4, 5, 6	10, 11, 12	13, 14, 15	05:00 to 07:00	1, 2, 3	7, 8, 9	13, 14, 15
	12:00 to 14:00	1, 2, 3	7, 8, 9	16, 17, 18	11:00 to 13:00	4, 5, 6	10, 11, 12	16, 17, 18
	17:00 to 19:00	4, 5, 6	10, 11, 12	13, 14, 15	19:00 to 21:00	1, 2, 3	7, 8, 9	13, 14, 15

Behavioral data of each minute were categorized into grazing and rumination, considering the main activity within each minute. Grazing was defined as cow’s muzzle being located near or above the grass and making a biting motion to ingest grass or chewing ripped grass with the head position down, or cow’s head position up and making a chewing motion to masticate the grazed grass. Alternatively, rumination was defined as regurgitation, chewing, salivation and swallowing of ingested grass (Bikker *et al*., 2014). The data were recorded on a manual spread sheet. Subsequently, the data were transferred manually to an electronic spread sheet (Microsoft Excel Version 2010; Microsoft Corporation, Redmond, WA, USA).

The MooMonitor+ was used for the automated data collection. It is a collar device on the cow’s neck containing a box with a 3-axis accelerometer was positioned on the right sight of the neck. This accelerometer measured activity in a 10 Hz resolution. On-board data analysis with a generic algorithm, which was identifying specific pattern for different categories such as rumination, grazing, resting, developed by Dairymaster, summarized activities occurring in the raw data into time spent at those activities for 15-min periods. These summarized periods were then transmitted wirelessly to a base station with a range of up to 2000 m. The base station is usually linked with the internet connection in a normal farm environment and corrected itself in time, based on a deviation of ±5 min. This time is also corrected on the sensors once they were in the range. To ensure correct positioning of the accelerometer box on the cow’s neck, a weight was applied at the lowest point of the collar. Cows within the group were identifiable by numbers painted on their sides.

#### Data preparation

The 1-min visually recorded data in the experimental dataset were summarized in 15-min and 1-h grazing and rumination periods to allow direct comparison with the data recorded by the automated method. The automatically captured data were classified into the categories of grazing and ruminating in 15-min summaries. Then four 15-min summaries were totaled to form 1-h summaries. Consequently, there were 504 15-min periods and 72 1-h periods of valid observations across the full database.

#### Statistical analysis

For the statistical analysis, R version 3.3.1 (R Foundation for Statistical Computing, Vienna, Austria) was used (statistical code see Supplementary Material S1). To assess agreement between numeric value data of the MooMonitor+ and visual observation, the Spearman’s Rank correlation (*r*_*s*_) and a concordance correlation coefficient (CCC) was calculated. The interpretation of *r*_*s*_-values and CCC were based on definitions by Hinkle (2003) as follows: Negligible = 0.0 to 0.3, low = 0.3 to 0.5, moderate = 0.5 to 0.7, high = 0.7 to 0.9 and very high = 0.9 to 1.00. Furthermore, the Bland–Altman analysis was applied to assess the agreement between visual observation and automated system. This was conducted in Microsoft Excel calculating the mean differences (bias; MooMonitor+ – visual observation) against the means of visual observation and MooMonitor+. The limits of agreement were calculated as ± 1.96×standard deviation from the mean difference. Although the parameters themselves were not normally distributed, the Bland–Altman analysis was used as the differences between the paired values did follow a normal distribution.

### Experiment 2

#### Animals and treatments

This experiment was conducted between 18 and 30 October 2016. A group of 12 cows was used in this study. The group of cows had an average of 255±11 DIM and was maintained in a herd of 55 cows in a spring calving dairy system. The cow group consisted of eight Jersey crossbred, three Holstein-Friesian and one Norwegian Red cows. There were three primiparous and nine multiparous cows, ranging from two to five lactations. Daily milk yield in the experimental period averaged 12.4±2.5 kg/day per cow and the average BW was 527±71 kg with a BCS of 2.9±0.1. The cow herd was milked twice daily and was away from pasture for ~2 h at each milking time, that is, 7:00 to 9:00 h and 15:00 to 17:00 h. All cows had a grass-based diet with an additional 2 kg concentrate offered per day. Grass height was measured daily with a rising plate meter (diameter 355 mm and 3.2 kg/m^2^). Pre-grazing height of pasture averaged 12.1±1.2 cm and post-grazing height averaged 4.1±0.2 cm. Grass quality was analyzed once weekly resulting in an average DM content of 16%, an average CP content of 27% and an average content of NDF and ADF of 41% and 23%, respectively. The average daily herbage allowance was 14.9±0.8 kg DM/cow per day during the experimental period.

#### Sensor technology and data collection

All 12 cows in the group were simultaneously equipped with the Moomonitor+ collar and the RumiWatch noseband sensor (Itin+Hoch GmbH, Liestal, Switzerland) to determine grazing and rumination time. The RumiWatch noseband sensor, integrated in a halter, has the capability to detect pressure peaks and classifies them into grazing or rumination behavior, such as grazing bites or rumination chews. In addition, the total time duration of those different classifications was recorded continuously. Raw data were recorded in a 10 Hz resolution. Further information about technical components can be found in Zehner *et al*. (2017). In the current study, the RumiWatch Manager 2 (V.2.1.0.0) was used to manage time synchronization and raw data recording of the devices. The RumiWatch Converter (V.0.7.3.36) was used for analyzing the raw data. Two recorded parameters of the RumiWatch halter were used to determine grazing time. They were EAT1TIME, which monitored grazing time with head position down. This included biting and chewing of ripped grass and EAT2TIME, which recorded grazing time with the head position up with chewing of grazed grass. Grazing time (referred to as EATTIME) was calculated as EAT1TIME+EAT2TIME. The internal time on the RumiWatch noseband sensors was synchronized to internet time (Coordinated Universal Time (UTC) +1 h) before the commencement of the experiment. A period of 2 days was allowed for adaption of the cows to the halter. The recording of one noseband sensor stopped after 6 days, therefore there were just 6 full days instead of 10 days included for one cow. The Moomonitor+ collar was applied at the start of the breeding season and the correct attachment of tag number and cow identification number was checked before the experiment. The time on the base station was synchronized and operated at UTC time.

#### Data preparation

The raw data of the RumiWatch noseband sensor were converted in 30-min summaries using the RumiWatch Converter V.0.7.3.36. The output of the MooMonitor+ was delivered in 15-min summaries and was totaled in 30-min summaries to allow comparison with the RumiWatch output. For analysis, the data were matched in an electronic spread sheet (Microsoft Excel Version 2010) with a time adjustment of −1 h for the RumiWatch data, due to the difference between summertime and UTC time. The 30-min values were then totaled to generate daily values. In total, there were *n* = 5579 valid observations at the 30-min resolution level, and *n* = 116 values at the daily level.

#### Statistical analysis

For statistical analysis the R version 3.2.2 (R Foundation for Statistical Computing) was used (statistical code see Supplementary Material S2). Comparison between the numerical data of the RumiWatch noseband sensor and the MooMonitor+ was analyzed using different statistical approaches.

**Figure 1:**
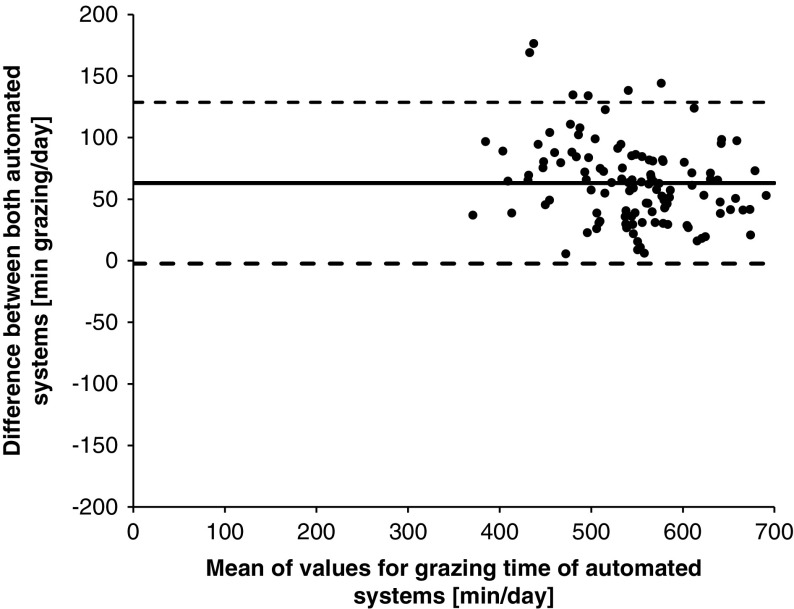
Agreement of MooMonitor+ collar and RumiWatch noseband sensor measurements of cow grazing time per day, displayed in a Bland–Altman Plot (solid line indicates the mean difference; dashed lines indicate upper and lower 95% limits of agreement), when grazing time was defined as EATTIME by the RumiWatch noseband sensor. EATTIME represents the sum of grazing time with head position down (EAT1TIME) and head position up (EAT2TIME).

For the analysis of 30-min summaries, the Anderson–Darling test was applied to evaluate if the data followed a normal distribution. Due to the data not being normally distributed, same statistical analysis and interpretation of results was used as in Experiment 1. The data summarized at a daily level followed a normal distribution based on the results of the Shapiro–Wilk normality test. Therefore, Pearson’s correlation coefficients (*r*) were calculated as well as the CCC-values. The values were interpreted by using the same categories as outlined above for the 30-min summaries. There was also a graphical analysis of the agreement with Bland–Altman Plots used to determine the agreement between the automated systems. This was conducted by plotting the differences (RumiWatch–MooMonitor+) against the means of RumiWatch and MooMonitor+. The Bland–Altman analysis indicated the mean difference (bias; solid line Figures 1, 3 and 4) between the paired automatically recorded values and their associated 95% limits of agreement, displayed as dashed lines in Figures 1, 3 and 4. The limits of agreement were calculated as ± 1.96×standard deviation from the bias.

**Figure 2:**
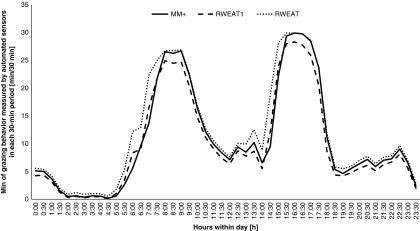
Graphical analysis of diurnal grazing time of cows defined by the MooMonitor+ (MM+) and by the RumiWatch noseband sensor with the parameter ‘RWEAT1’, which represents the grazing time with head position down (EAT1TIME) and with the parameter ‘RWEAT’, which represents the sum of grazing time with head position down (EAT1TIME) and head position up (EAT2TIME) averaged in 30-min periods.

**Figure 3:**
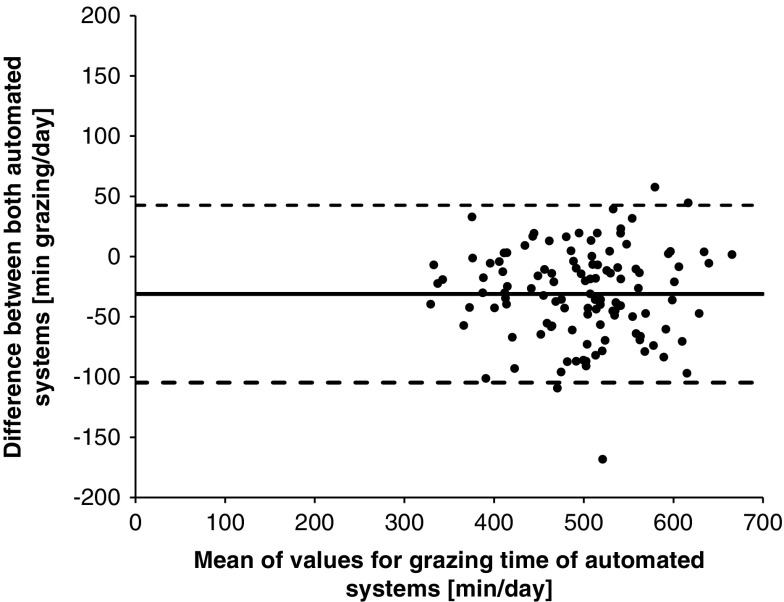
Agreement of MooMonitor+ collar and RumiWatch noseband sensor measurements of cow grazing time per day, displayed in a Bland–Altman Plot (solid line indicates the mean difference; dashed lines indicate upper and lower 95% limits of agreement), when grazing time was defined as EAT1TIME by the RumiWatch noseband sensor. EAT1TIME represents the grazing time with head position down.

## Results

### Experiment 1

The results of the comparison between visual observation and automated measurement are presented in Table 2. Median grazing time of 12 min/15 min and 38 min/h were recorded by visual observation. Alternatively, median grazing time of 12 min/15 min and 37.5 min/h were recorded by the automated sensor. However, overall grazing time was slightly overestimated by the automated measurement compared to visual observation. This fact is observed in mean differences of 0.27 min/15 min, and 1.0 min/h. The correlations between the paired measurements were very high with *r*_*s*_ = 0.90 and CCC = 0.95 for 15-min summaries and *r*_*s*_ = 0.94 and CCC = 0.97 for 1-h summaries.

**Table 2:** Spearman’s rank correlation coefficient (*r*_*s*_), concordance correlation coefficient (CCC) and Bland–Altman analysis (bias, upper and lower 95% limits of agreement) of cow grazing and rumination data, recorded by visual observation and by automated measurements in 15-min and 1-h resolutions

Behaviors	*r*_*s*_	CCC	Bias	Lower	Upper
Grazing time (min/15 min)	0.90	0.95	0.27	−3.76	4.31
Rumination time (min/15 min)	0.93	0.98	0.10	−2.09	2.30
Grazing time (min/h)	0.94	0.97	1.01	−10.18	12.21
Rumination time (min/h)	0.97	0.98	0.00	−4.93	4.93

However, the correlation between visually captured data and automatically recorded data was higher for rumination time than grazing time at both the 15-min and 1-h resolutions. Due to a small proportion of time associated with rumination in the observation periods, median rumination times of 0 min/15 min and 8 min/h for rumination time were recorded by visual observation. Alternatively, median rumination times of 0 min/15 min and 6.5 min/h were recorded by the automated sensor. For rumination time, the correlation between paired measurements by visual observation and automated recording may be described by *r*_*s*_ = 0.93 and CCC = 0.98 for 15-min summaries. This was weaker than for the 1-h summaries with *r*_*s*_ = 0.97 and CCC = 0.98.

### Experiment 2

The correlation of grazing time measured by the MooMonitor+ and the RumiWatch noseband sensor in 30-min summaries was very high. An *r*_*s*_-value of 0.91 and a CCC-value of 0.95 were observed. Median grazing times of 3.5 min/30 min and 5.8 min/30 min were recorded by the MooMonitor+ collar and the RumiWatch noseband sensor, respectively. In analysis of daily values, grazing time averaged 513±75 min/day with the MooMonitor+ and 576±66 min/day with the RumiWatch. The accordance of grazing time per day between the two automated systems was analyzed using a Bland–Altman Plot (Figure 1). The 95% limits of agreement ranged between −2 and 129 min/day. In addition, a mean bias of 63 min/day was observed indicating a higher value of grazing time measured by the RumiWatch in comparison to the MooMonitor +. This higher value of grazing time was also captured in a lower Pearson’s correlation coefficient of 0.89 and a CCC of 0.63 in the daily summaries.

When examining grazing time of MooMonitor+ and RumiWatch above in terms of correlation in 30-min summaries and total grazing time per day, this time was a measurement of the sum of EAT1TIME and EAT2TIME. This represented biting and chewing (head position down) and chewing the grazed grass (head position up). The graphical analysis in Figure 2 demonstrated an overestimation by the RumiWatch when using the parameters EAT1TIME+EAT2TIME.

Alternatively, when the EAT1TIME parameter was used, it only focused on grazing behavior with head position down (biting and chewing) and this resulted in a median value of 3.1 min/30 min. In this scenario, the correlation between the automated sensor systems was higher with an *r*_*s*_-value of 0.94 and a CCC of 0.98 for 30-min resolutions.

Daily grazing times recorded by the RumiWatch noseband sensor, using the EAT1TIME averaged 482±75 min/day compared to 576±66 when the sum of EAT1TIME and EAT2TIME was used. This relationship between the MooMonitor+ and the RumiWatch (considering EAT1TIME as grazing time per day) is shown in Figure 3. The RumiWatch parameter EAT1TIME recorded a lower grazing time compared to MooMonitor+, captured with a mean bias of −31 min/day and the 95% limits of agreement ranged from −105 to 43 min/day. However, comparing daily grazing time recorded by the MooMonitor+ to the RumiWatch noseband sensor when EAT1TIME was used increased the CCC-value to 0.80, but decreased the Pearson’s *r* slightly to *r* = 0.87 for daily grazing time.

The data captured by the MooMonitor+ and the RumiWatch showed a higher accordance in detecting rumination time. Rumination time was recorded at a 30-min resolution. Median rumination times of 3.8 min/30 min and 3.6 min/30 min were recorded by the MooMonitor+ collar and by the RumiWatch noseband sensor, respectively. The correlation with *r*_*s*_-value of 0.96 and a CCC-value of 0.99 highlight the high accordance between the MooMonitor+ and the RumiWatch noseband sensor in detecting rumination based on 30-min summaries.

**Figure 4:**
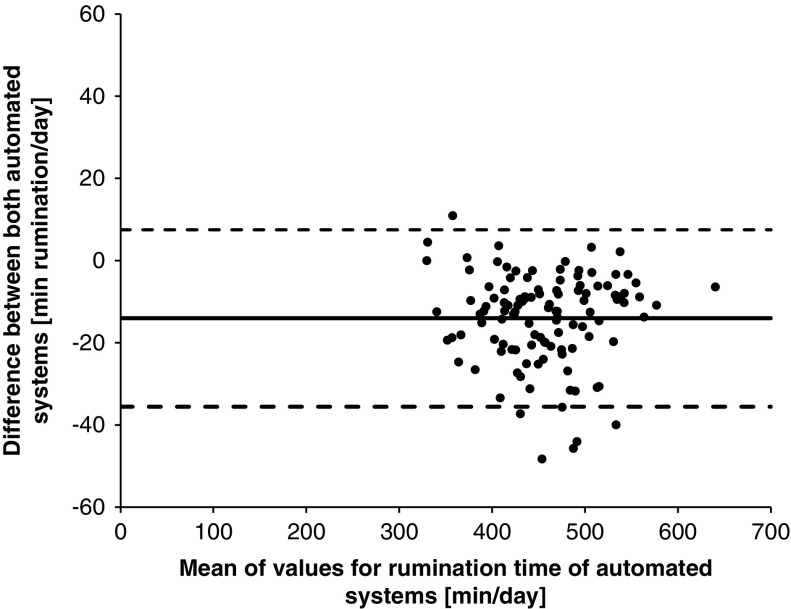
Agreement of MooMonitor+ and RumiWatch noseband sensor measurements of cow rumination time per day, displayed in a Bland–Altman Plot (solid line indicates the mean difference; dashed lines indicate upper and lower 95% limits of agreement).

In the analysis of the daily values, rumination time averaged 463±58 min/day for the MooMonitor+ and 407±57 min/day for the RumiWatch. The MooMonitor+ measured a slightly higher rumination time compared to the RumiWatch, which may be observed in Figure 4 with a negative mean bias of −14 min/day. In addition, the 95% limits of agreement ranged between -35.5 and 7.5 min/day. However, there is a very high correlation between both systems in recording daily rumination time with a Pearson’s *r*-value of 0.98 and a CCC-value of 0.95.

## Discussion

The comparison between visual observations and automated measurements of grazing time showed a very high correlation in Experiment 1. The results demonstrated a higher correlation compared to other commercial sensors with an *r*_*s*_-value of 0.90 and CCC = 0.95 for a 15-min resolution and *r*_*s*_-value of 0.94 and CCC = 0.97 for a 1-h resolution. For example, in a study by Borchers *et al*. (2016), an *r* = 0.88 and CCC = 0.82 was established for the CowManager ‘SensOor’ system (Agis, Harmelen, The Netherlands) and *r* = 0.93 and CCC = 0.79 for the ‘Track A Cow’ system (ENGS, Rosh Pina, Israel). In contrast to the current study, those systems were validated by measuring feeding time of housed dairy cows with a total-mixed ration fed twice daily. There are some sensors available to measure grazing behavior with a high accuracy for research purposes, for example, the IGER system, a microcomputer-based system for digital recording of jaw movements, for determining grazing time and grazing bites (Rutter *et al*., 1997), or the Lifecorder Plus for measuring grazing time and pattern used in an experiment of Delagarde and Lamberton (2015). Any of these mentioned sensor systems were not applied on a larger scale on commercial farms. However, a study by Molfino *et al*. (2017) investigated the accuracy of a commercially applied sensor compared to visual observation with results of CCC = 0.99 and 0.80 for measured grazing and rumination time, respectively.

The measurement of rumination time was more accurate than the measurement of grazing time by the MooMonitor+ when compared against visual observation with a very high correlation of *r*_*s*_-value of 0.93 at 15-min resolution, and *r*_*s*_-value of 0.97 at 1-h resolution. Those values are comparable with the Hi-Tag rumination monitoring system (SCR Engineers Ltd, Netanya, Israel), which was validated against visual observation in the study of Schirmann *et al*. (2009) showing an *r*-value of 0.93. In contrast to this result Elischer *et al*. (2013) found that a rumination collar with an integrated microphone, which was used in an automatic milking system on pasture, had a moderate correlation against visual observation with an *r*-value of 0.65. Those findings were explained by possible malfunction of the microphone or improper placement of the sensor on the cow’s neck. In the current study, similar issues with the MooMonitor+ were not observed.

The correlation between the MooMonitor+ and the RumiWatch was lower for grazing than for rumination time. The RumiWatch noseband sensor recorded a higher value of grazing time. This prompted some more detailed analysis to be conducted in comparing the grazing time of the MooMonitor+ against the EAT1TIME recording parameter of the RumiWatch, which represented recording of grazing time with the head position down. This comparison showed higher correlations with *r*_*s*_ = 0.94 and a CCC of 0.98 for the 30-min resolution and a smaller mean difference between the two systems. This was presumably caused by exclusion of time recorded by the EAT2TIME parameter. On further investigation it was established, that if a cow was walking, the sensitive noseband pressure sensor was recording a portion of feeding time, due to the head movement while walking. This effect was eliminated by using just the parameter EAT1TIME. The described movement effect could not be identified in an indoor environment, which was the primary environment for the development of the RumiWatch noseband sensor. This issue may be addressed in further development by applying new algorithms for analyzing grazing behavior of dairy cows in a new RumiWatchConverter. Nevertheless, the accuracy of the MooMonitor+ against the RumiWatch noseband sensor was very high.

The results demonstrated that the MooMonitor+ is an accurate measurement tool for monitoring grazing behavior of cows in a 15-min data resolution. Other studies have also identified different sensors validated for measuring various grazing parameters. A selection of these are presented in Table 3. This information is based on an extensive literature analysis in which the application of different sensors for research purposes or commercial use was examined. Similar criteria may be applied to the sensors used in the current study to assess their attributes for research or commercial situations. Measurement of grazing bites and chews as well as the combined ‘chewbites’ using acoustic measurements is presented in studies by Navon *et al*. (2013) and Milone *et al*. (2012). Both systems were limited to grazing jaw movements and therefore rumination chews were not detected. The system validated by Navon *et al*. (2013) focused on grazing jaw movements showed a high accuracy and may be feasible to use for research applications. Nevertheless, the commercial application is limited as the data analysis is very laborious and the attachment of the sensor in middle of the animal’s forehead with rubberbands is not practical. In grazing behavior research, the IGER behavior recorder was used intensively in various studies. It consisted of a jaw movement sensor and a datalogger and was able to measure rumination and grazing times as well as rumination chews and grazing bites/chews. The raw data were analyzed via an associated software (Rutter, 2000). Alternatively, the parameters of grazing bites and chews were validated against visual observation but were reported as being difficult to distinguish in a study by Champion *et al*. (1997). However, this technology and data collection is outdated and the production of the dataloggers has been discontinued. The RumiWatch noseband sensor, compared to the MooMonitor+ in the current study, may be considered as a more advanced technology than the IGER behavior recorder due to longer data recording periods and a more simplified application and data analysis. Rombach *et al*. (2018) conducted a comprehensive validation study on grazing and supplemented cows to evaluate the accuracy of the RumiWatch noseband sensor. There was also the performance of a newly developed analysis software investigated, with an improved accuracy on measuring grazing behavior parameters such as total number of eating chews, number of rumination chews and times spent engaging in these activities. A validation study by Werner *et al*. (2017), in which a subsequent version of the analysis software used in the study of Rombach *et al*. (2018) was used, also demonstrated a high accuracy in measuring different parameters of cows’ grazing behavior, such as rumination time and chews as well as grazing time. The parameters of grazing bites as well as grazing bouts and rumination bouts are solely measured by the newest algorithms in the RumiWatch Converter. These measurements were also proven to be very high in accordance with the visual observation based on the study of Werner *et al*. (2017).

**Table 3:** A selection of validation studies to measure grazing behavior of cows, combined with an assessment of feasibility for research purposes (R) or commercial application on farms (F)

Measured parameters	Technology used	Resolution as listed in reference	Assessment^1^	References
Foraging, resting and walking	Global Positioning System (GPS) collar	1 min sampling	R: + F: −−	Spink *et al*. (2013)
Grazing jaw movements	Wireless microphone	1 min recording, 10 min analysis	R: + F: −−	Navon *et al*. (2013)
Bites, chews and compound chewbites	Wireless microphone	13 min	R: − F: −−	Milone *et al*. (2012)
Grazing, ruminating or other activities	Jaw movement sensor	Continuous measurement for 24 h	R: + F: −−	Rutter *et al*. (1997), Rutter (2000)
Grazing bites and chews	Jaw movement sensor	Continuous measurement for 24 h	R: − F: −−	Champion *et al*. (1997)
Grazing, ruminating or other activities, grazing bites and chews, rumination chews, grazing and rumination bouts	Noseband pressure sensor	Grazing/rumination times: 1 min and 1 h resolution Grazing bites/rumination chews 5 min resolution	R: ++ F: −	Werner *et al*. (2017)
Grazing time	Accelerometer	4-s sampling periods or preprocessed data 2 min	R: + F: −−	Delagarde and Lamberton (2015)
Grazing bites	Accelerometer	Storage 1 day or 1 h on the pedometer	R: + F: −−	Umemura (2013)
Rumination activity	Cow rumination monitors	2 h blocks	R: − F: −	Elischer *et al*. (2013)
Grazing, ruminating and resting	Accelerometer	Raw data: 1 s intervals Analysis of 1-min resolution data	R: + F: −−	Decandia *et al*. (2017)
Grazing, resting and ruminating	Accelerometer	1-min intervals	R: ++ F: ++	Molfino *et al*. (2017)
Grazing time	Accelerometer	15-min periods	R: + F: ++	Ipema (2015)

++ = highly suitable;+ = partial suitable; − = partial usable; −− = not applicable.

1 The assessment results are based on accuracy in measured parameters and applicability.

However, most studies apply accelerometers to measure different parameters of grazing behavior. However, as shown in Table 3, there are different approaches to record and use the acceleration data. The position of the accelerometer on a cow may be attached to a halter (Decandia *et al*., 2017) or a neck collar (Umemura, 2013), and the measured parameters may vary, for example, grazing time or grazing bites. Studies by Molfino *et al*. (2017) and Ipema (2015) investigated the accuracy of commercially applicable sensors and showed a high accuracy in measuring grazing time in both studies and rumination time in the study of Molfino *et al*. (2017).

Focusing on comparing both sensor systems of the current study in terms of their application potential and requirements for each focal group in using those sensors, the purpose of the systems must be considered. The main purpose of the MooMonitor+ is usage and support in decision-making on commercial dairy farms, whereas the RumiWatch system was mainly designed for research purposes as a high precision measurement sensor. The RumiWatch noseband sensor has the ability to detect and record each individual jaw movement of the cow. This high-resolution information is particularly important when using the device for investigating research questions as researchers need a very detailed measurement of cow behavior. Contrary to this, from a farmer’s perspective it is not feasible for daily use in a commercial farm environment, as its integration into a halter is not as practical as in a collar. Also, the extensive amount of data that may be collected is not required by a farmer, as the farmer generally wishes to just monitor his animals and might want to get some easy-to-use decision support in terms of health, breeding or grazing issues as herd sizes increase. These requirements might be better fulfilled by the MooMonitor+. It is still able to measure grazing behavior on a high resolution with 15-min summaries, but does summarize analyzed data into clear information to the farmer, which will be communicated via an application on the smartphone. This does include, for example, alerts, when an individual cow’s behavior is deviating from the herds behavior. Thus, since the research and farming communities have different requirements, both the MooMonitor+ and the RumiWatch can address these requirements through their different measurement approaches.

## Conclusion

The MooMonitor+ collar indicated a very high correlation when measured against visual observation and the RumiWatch noseband sensor. The recording of rumination time by the MooMonitor+ had a greater agreement with both visual observation and the RumiWatch sensor than the recording of grazing time. However, the correlation of detecting grazing time with visual observation and the RumiWatch was also very high. Considering the MooMonitor+ as a sensor technology for measuring grazing behavior, it has a number of benefits for commercial use on farms. The farmer gets an easy-to-use, robust, long-lasting device with a very high accuracy in measuring grazing behavior on a daily basis.

Depending on the research question, the MooMonitor+ collar can be used for scientific purposes. However, it must be recognized that detailed information about the grazing behavior in 1-min resolutions cannot be detected with the device in its current format. Thus, in further sensor technology development, it is crucial to consider the main focus of the application potential and try to address the requirements for each focal group. Whereas the volume and detail of data should be very high for researchers, the farmer does not require a similar level of data detail. A precise and distinct usage of the sensor, summarizing analyzed information with defined action points by the decision support tool is mainly what should be aimed for in regard to the application on farms.
