# Supplementary material for: Evaluation and application potential of an accelerometer-based collar device for measuring grazing behavior of dairy cows
Source: Animal. 2019 Feb 8;13(9):2070–9. doi: 10.1017/S1751731118003658 (PMC6700710; doi:10.1017/S1751731118003658)
Supplement: Supplementary file 1 [file S1751731118003658sup001.docx]

Evaluation and application potential of an accelerometer-based collar device for measuring grazing behavior of dairy cows

J. Werner, C. Umstatter, L. Leso, E. Kennedy, A. Geoghegan, L. Shalloo, M. Schick, B. O’Brien

animal

The International Journal of Animal Biosciences

**Supplementary Material 1 (S1)**

############################

##### loading packages #####

require(epiR)

citation(package="epiR")

require(BlandAltmanLeh)

citation(package="BlandAltmanLeh")

require(hydroGOF)

citation(package="hydroGOF")

require(MethComp)

citation(package="MethComp")

require(nortest)

citation(package="nortest")

setwd("D://Teagasc_Work//Jessica//Experiments//DF//Validation_MM_final_DF")

getwd()

#################### ##### MMvsVis ######

#### CREATING DFs

DF_MM_Valid_15Min <-read.table("DF_MM_Valid_15Min.csv", header=TRUE, sep=",", dec=".")

str(DF_MM_Valid_15Min)

##### RW vs MM_15Min #######

### RumiTime ###

attach(DF_MM_Valid_15Min)

cor.test(Ruminating,Rumination_MM)

cor.test(Ruminating,Rumination_MM,method="spearman", exact=FALSE)

epi.ccc(Ruminating,Rumination_MM, ci = "z-transform", conf.level = 0.95)

bland.altman.plot(Ruminating,Rumination_MM, two = 1.96, mode = 2,

graph.sys = "base", conf.int = 0.95, silent = FALSE, sunflower = FALSE,

geom_count = FALSE)

### Feeding####

str(DF_MM_Valid_15Min)

attach(DF_MM_Valid_15Min)

cor.test(Grazing, Feeding_MM,method="spearman", exact=FALSE)

epi.ccc(Grazing, Feeding_MM, ci = "z-transform", conf.level = 0.95)

bland.altman.plot(Grazing, Feeding_MM, two = 1.96, mode = 2,

graph.sys = "base", conf.int = 0.95, silent = FALSE, sunflower = FALSE,

geom_count = FALSE)

detach(DF_MM_Valid_15Min)

##### RW vs MM_1h #######

### RumiTime ###

DF_MM_Valid_1h <-read.table("DF_MM_Valid_1hour.csv", header=TRUE, sep=",", dec=".")

attach(DF_MM_Valid_1h)

str(DF_MM_Valid_1h)

cor.test(Ruminating,Rumination_MM)

cor.test(Ruminating,Rumination_MM,method="spearman", exact=FALSE)

epi.ccc(Ruminating,Rumination_MM, ci = "z-transform", conf.level = 0.95)

bland.altman.plot(Ruminating,Rumination_MM, two = 1.96, mode = 2,

graph.sys = "base", conf.int = 0.95, silent = FALSE, sunflower = FALSE,

geom_count = FALSE)

### Feeding####

str(DF_MM_Valid_1h)

attach(DF_MM_Valid_1h)

cor.test(Grazing, Feeding_MM,method="spearman", exact=FALSE)

epi.ccc(Grazing, Feeding_MM, ci = "z-transform", conf.level = 0.95)

bland.altman.plot(Grazing, Feeding_MM, two = 1.96, mode = 2,

graph.sys = "base", conf.int = 0.95, silent = FALSE, sunflower = FALSE,

geom_count = FALSE)

detach(DF_MM_Valid_1h)

**Supplementary Material 2 (S2)**

############################

##### loading packages #####

require(epiR)

citation(package="epiR")

require(BlandAltmanLeh)

citation(package="BlandAltmanLeh")

require(hydroGOF)

citation(package="hydroGOF")

require(MethComp)

citation(package="MethComp")

require(nortest)

citation(package="nortest")

#### CREATING DFs

########## 30Min_MM_vs_RW ##########

#HOME PC/INTENSO

setwd("D://Teagasc_Work//Jessica//Experiments//MM_Validation//2nd_period_autumn")

getwd()

#### CREATING DFs

DF_MM_30 <-read.table("Dataset30Min.csv", header=TRUE, sep=",", dec=".")

attach (DF_MM_30)

str(DF_MM_30)

### Test for normality with Anderson-Darling test for normality ###

library(nortest)

ad.test (RUMINATETIME)

ad.test (EAT1TIME)

ad.test (EATTIME )

#### Spearmans Rank correlation ###

###Feeding###

cor.test(EAT1TIME, FEEDING,method="spearman", exact=FALSE)

cor.test(EATTIME, FEEDING,method="spearman", exact=FALSE)

###Rumination###

cor.test(RUMINATETIME, RUMUNATION,method="spearman", exact=FALSE)

##### Concordance Correlation Coefficient####

epi.ccc(RUMINATETIME, RUMUNATION, ci = "z-transform", conf.level = 0.95)

epi.ccc(EAT1TIME, FEEDING, ci = "z-transform", conf.level = 0.95)

epi.ccc(EATTIME, FEEDING, ci = "z-transform", conf.level = 0.95)

detach (DF_MM_30)

setwd("D://Teagasc_Work//Jessica//Experiments//DF//Validation_MM_final_DF")

getwd()

########## DAILY_MM_vs_RW ##########

DF_MM_24 <-read.table("DF_MM_RW_Daily.csv", header=TRUE, sep=",", dec=".")

attach (DF_MM_24)

str(DF_MM_24)

### Test for Shapiro-Wilk Normality Test

shapiro.test(DIFF_RUMI)

shapiro.test (DIFF_EAT1)

shapiro.test (DIFF_EAT)

shapiro.test(EATTIME)

shapiro.test (EAT1TIME)

shapiro.test (RUMINATETIME)

shapiro.test(FEEDING)

shapiro.test (RUMUNATION)

##### PEARSON'S R####

###Feeding###

cor.test(EAT1TIME, FEEDING)

cor.test(EATTIME, FEEDING)

###Rumination###

cor.test(RUMINATETIME, RUMUNATION)

##### Concordance Correlation Coefficient####

epi.ccc(RUMINATETIME, RUMUNATION, ci = "z-transform", conf.level = 0.95)

epi.ccc(EAT1TIME, FEEDING, ci = "z-transform", conf.level = 0.95)

epi.ccc(EATTIME, FEEDING, ci = "z-transform", conf.level = 0.95)

bland.altman.plot(EATTIME, FEEDING, two = 1.96, mode = 2,

graph.sys = "base", conf.int = 0.95, silent = FALSE, sunflower = FALSE,

geom_count = FALSE)

detach(DF_MM_24)
